# Supplementary material for: Functional identification of Annexin B1 and Annexin B2 from Cysticercus cellulosae and their mechanism in plasma membrane repair
Source: PLoS Negl Trop Dis. 2025 Apr 17;19(4):e0013015. doi: 10.1371/journal.pntd.0013015 (PMC12005505; doi:10.1371/journal.pntd.0013015)
Supplement: Attachment — Submitted filename: point-by-point response.pdf [file pntd.0013015.s011.pdf]

Dear Editors and Reviewers:

Thank you for your letter and for the reviewers' comments concerning our manuscript entitled "Functional Identification of Annexin B1 and Annexin B2 from *Cysticercus cellulosae* and Their Mechanism in Plasma Membrane Repair" (ID: PNTD-D-24-01289). We are very excited to receive your letter, especially when we see that three reviewers have provided valuable suggestions for our manuscript. Those comments are all valuable and very helpful for revising and improving our paper, as well as the important guiding significance to our researches. We feel that our work has been valued. And if we can revise all the experts' suggestions in place, we believe that our work and manuscript content will make significant progress. At the same time, this is also a challenge for us. We have studied comments carefully and have made correction which we hope meet with approval. If there are any shortcomings in our modifications, please feel free to contact us at any time.

We sincerely thank the editor and all reviewers for their valuable feedback that we have used to improve the quality of our manuscript. The reviewer comments are laid out below in italicized font and specific concerns have been numbered. Our response is given in normal font. The manuscript has been modified/added to using tracked changes/highlighted in yellow.

Reviewer #1

[Suggestion 1]: *English writing is not fluent, requring major revision.*

[Reply]: Thank you for your suggestions. We have made some modifications and will continue to work hard on our English writing. If there is anything else that is inappropriate, please let us know in a timely manner, and we would be very grateful.

[Suggestion 2]: *Line 158 Expression of Annexin B1 and Annexin B2. Plese provide genes' IDs or sequence used.*

[Reply]: Thank you for your comments. We have now supplemented this part. Specific content can be found in lines 177-178.

[Suggestion 3]: *Line 26, "coli" should be italic.； Line 160: "Escherichia coli" should be read as italic.； Line 179: "B" should be lower case.*

[Reply]: We have made correction according to the Reviewer's comments.

[Suggestion 4]: *Figures 3 and 4 can be combined into one figure.*

[Reply]: We combined the figures according to your opinion.

[Suggestion 5]: *Line 428, "+Ca^{2+} no repair group of Annexin B2.", is this sentence correct?*

[Reply]: Sorry, this was an error in our drafting. Thank you for pointing it out.

Reviewer #2

[Suggestion 1]: *Figure 4, no scale bar.*

[Reply]: Sorry about that. There is a scale in the lower right corner of each small picture. However, due to the low clarity of the picture, the reviewer may overlook it. We have adjusted the resolution of the picture to solve this problem.

[Suggestion 2]: *Figure 5, provide raw data and details on statistical analysis. There are only two levels of significance either ns or $P < 0.0001$ (****) that seems possible although improbable.*

[Reply]: Thank you for your meticulous review of the manuscript. We have added a new control group in the experiment and reanalyzed all the data using one-way ANOVA. We have also redrawn the figures accordingly.

[Suggestion 3]: *Figure 8. Repair of plasma membrane after transfection. Should be Repair after the injury.*

[Reply]: We changed it to 'The plasma membrane repairs itself after injury following transfection'.

[Suggestion 4]: *Figure 9. Fluorescence intensity quantization of FM1-43 in cells. Example of real time imaging of cell membrane repair in response to laser injury from "[29] Sønder SL, Ebstrup ML, Dias C, Heitmann ASB, Nylandsted J. Plasma Membrane 658 Wounding and Repair Assays for Eukaryotic Cells. Bio Protoc. 2022;12(11):e4437. 659 doi:10.21769/BioProtoc.4437" has two-fold difference in F/F0 in control cells with and without Ca2+ because of the divalent-membrane interaction itself. Authors may want to discuss why there is no Ca2+ effect in control cells in their control experiment. FM1-43 kinetics supposed to show saturation upon membrane repair. Authors can discuss why they see near linear dye accumulation.*

[Reply]: It seems that the reviewer has read our manuscript in great detail. Considering that other readers may have similar questions, we have discussed the potential reasons in the Discussion section. Specific content can be found in lines 605-618.

[Suggestion 5]: *Methods section was going to use (AUC) analysis followed by unpaired t-test with Welch's that appears missing from Figure 9.*

[Reply]: I think the reviewer might have misunderstood my intention. It wasn't that we used two different methods to obtain two separate results. Instead, we first calculated the AUC for each curve, but at this stage, we couldn't yet draw conclusions about significant differences between groups. Only after applying the Welch's unpaired t-test to the AUC values did we arrive at our final conclusions regarding significance. Therefore, we are not actually missing any figures.

[Suggestion 6]: *Authors may want to extend and clarify figures captions.*

[Reply]: Thank you for your suggestions. We have made significant revisions to the figures. If there are any further issues, please let us know promptly.

[Suggestion 7]: *The statistical test performed for the anticoagulant activity's measurement requires additional transparency and possible reevaluation. Another discrepancy is a lack of Ca2+ effect on the cell membrane repair in control cells and lack of saturation of the FM1-43 dye implies lack of membrane repair but rather continuous dye uptake by the damaged cells. The new experiment author may want to consider is to replicate the FM1-43 dye protocol in control conditions or discuss the observed discrepancies.*

[Reply]: Regarding the coagulation function experiments, we have added a new control group and reanalyzed the data. The second question you raised appears to be similar to [suggestion 4]. We have discussed the potential reasons for this issue in the Discussion section. Specific content can be found in lines 605-618.

Reviewer #3

[Suggestion 1]: *Line 32: The immunohistochemistry results indicate the localization of annexin B1 and B2 but do not provide direct evidence supporting their proposed functions, such as maintaining the worm's integrity, assisting movement, and nutrient absorption and digestion. Additional functional assays will be required to substantiate these claims.*

[Reply]: We deleted the unconfirmed speculation according to your suggestion.

[Suggestion 2]: *Line 40: Similar concerns apply to the interpretation of the plasma membrane repair test. The study should clarify the structural and compositional similarities and differences between the parasite's plasma membrane and mammalian ones. How do these factors influence the proposed membrane repair function of annexin B1 and B2 in Cysticercus cellulosae?*

[Reply]: Thank you for your suggestions. In fact, we regard mammalian cells as a model. To clarify the similarities and differences in the structure and composition of the plasma membranes of these two species, it is not a small experiment. In detail, thin-layer chromatography or high-performance liquid chromatography can be used to compare the types and contents of membrane lipids; gas chromatography-mass spectrometry can be employed to determine the types, chain lengths, and degrees of unsaturation of fatty acids; mass spectrometry can be utilized to compare the differences in key membrane proteins between the two; electron microscopy can be used to observe the membrane thickness, morphology, and surface characteristics of both; and the permeability of the membranes and the functions of membrane transport proteins can be studied, among other things.

[Suggestion 3]: *Line 44: The claim that annexin B1 and B2 contributes to immune evasion or inhibition of host immunity lacks direct supporting evidence. The authors should specify whether these proteins have multiple functions, including structural maintenance, facilitation of parasite migration, nutrient digestion and absorption, and immune evasion and provide experimental validation or references to support each role.*

[Reply]: We have revised the conclusions in accordance with the reviewer's suggestions. Specific content can be found in lines 43-47.

[Suggestion 4]: *The introduction should provide a clear rationale for why the authors specifically pursued the anticoagulant and plasma membrane repair functions of annexins based on prior studies and literature. If certain findings are not novel, the authors should cite papers and acknowledge prior work accordingly.*

[Reply]: Thank you for your suggestions. We have revised the introduction, refined the achievements of our predecessors, and clarified the motivations for our research. Specific content can be found in lines 141-149.

[Suggestion 5]: *Line 51: This statement is logically flawed. The fact that a disease is classified as "neglected" does not imply that the pathogen has exceptional immune*

evasion abilities. In other words, a pathogen's ability to evade the host immune system is not a direct consequence of the disease itself. The authors should rephrase this statement with supporting evidence and references.

[Reply]: I'm sorry that my words have caused you trouble. To clarify, there is no causal relationship between 'neglected diseases' and 'strong immune evasion ability'. We have revised the wording accordingly. Specific content can be found in lines 80-81.

[Suggestion 6]: *Line 79: The authors state that the mechanisms of invasion and immune escape are not well understood, yet the rest of paragraph describes how the parasites modulate the host immune response for survival in the hosts. The contradiction needs to be addressed, either by clarifying the unknown aspects of immune evasion or refining the statement to acknowledge existing knowledge.*

[Reply]: Thank you for your advice. We dealt with the contradiction.

[Suggestion 7]: *Line 107: The degree of conservation of the proteins should be clarified. Are the authors referring to functional conservation, sequence conservations, or both? What is the level of conservation, across eukaryotes, or more broadly prokaryotes and eukaryotes?*

[Reply]: Sorry, our expression is not clear enough. We have corrected it to 'highly conserved sequence'.

Regarding the degree of conservation in Annexin structure, in which species is it conserved? We have addressed this in the article and provided our answer 'Any member of the Annexin family'.

[Suggestion 8]: *Line 110: The phrase "the unique and highly conserved C-terminal" is contradictory. If a sequence is highly conserved, it implies similarity across species or homologs, whereas "unique" suggests distinctiveness. The authors should clarify whether they mean that the C-terminal is conserved within the annexin family while having functional specificity.*

[Reply]: Sorry, our expression is not clear enough. We have corrected it

All annexins possess a common folding motif, the C-terminal "core domain" (see Moss SE et al. Genome Biol. doi: 10.1186/gb-2004-5-4-219). The core domain incorporates functional sequences that are common for all members of the annexin protein family including Ca^{2+}-binding sites and sites responsible for the interactions with negatively charged phospholipids. In contrast, the NH2-terminal domains are variable and are thought to confer specific properties of each individual annexin (see

Gerke V et al. Phusiol Rev. doi: 10.1152/physrev.00030.2001.). At least 3 of the four Annexin repeat units are highly conserved in animals, while only 1 or 2 in plants (usually the 1st and 4th replicates) are conserved (see Hofmann, A. Annexins in the plant kingdom: perspectives and potentials. Annexins.; Gerke V et al. Nat Rev Mol Cell Biol. doi:10.1038/nrm1661).

[Suggestion 9]: *Line 126: The authors should specify how the expression of ANX A1 protein in parasitic vacuoles supports its potential as a therapeutic target. Additional supporting evidence or mechanistic details are needed to justify this claim.*

[Reply]: We removed this sentence without additional evidence to replace it with new content. Specific content can be found in lines 127-128.

[Suggestion 10]: *Lines 146-154: The overall conclusion is not well-supported by observations and/or data presented in the study. The authors make broad claims about annexin B1 and B2's roles in parasite invasions and immune evasions without providing sufficient experimental validation. In addition, the section lacks coherence, as it presents multiple functional roles without a clear logical connection between them. The authors should refine their conclusions to align more closely with the data presented and clarify the mechanistic links between annexin functions.*

[Reply]: Thank you for your suggestion. We have incorporated additional content into this section to elaborate that our speculations still require direct evidence, which makes them appear more scientific and rigorous.

[Suggestion 11]: *Lines 56-57: These are example sentences which need revisions. Sentences should not begin with "And".*

[Reply]: Thanks. We have made correction according to the Reviewer's comments.

[Suggestion 12]: *Line 156: The "Study animals" subsection only says "2 ±0.2 kg adult female rabbits." The authors should provide more details such as the rabbits' age, housing conditions, feeding regimen, and ethical approvals for animal use. Furthermore, while the study includes pig samples, the manuscript does not describe their source, handling, or relevant experimental details. These omissions need to be addressed to ensure transparency and reproducibility.*

[Reply]: We have added more details about the test animals. Specific content can be found in lines 172-174.

[Suggestion 13]: *Line 158 (Expression of annexin B1 and annexin B2): Where were the target gene sequences retrieved from? Is there any transcriptomic evidence that there may be multiple isoforms of the genes? What primers were used? What was the template material (e.g., total RNA or cDNA) for amplification? Where were the worms sourced from? How many worms were used?*

[Reply]: Sorry, this is our oversight. We have added the GenBank number of the target sequence. The template material we used is cDNA stored in pre-laboratory frozen. As for the more detailed aspects of the protein expression process, these are not technical issues that warrant extensive discussion (see Song H, et al. Parasit Vectors. doi:10.1186/s13071-021-04596-7.; Guo C, et al. Genes (Basel). doi:10.3390/genes9110559.; Tararam CA, et al. Exp Parasitol. doi:10.1016/j.exppara.2010.04.008.; Song X, et al. Trop Med Hyg. doi:10.4269/ajtmh.15-0452). Protein expression technology is now highly mature and diverse, and is widely used in most laboratories.

[Suggestion 14]: *Line 173 (Purification of annexin B1 and annexin B2): The group without IPTG was used as a negative control, but why wasn't the template empty pET-28a(+) vector (without the insert) or a vector with a gene insert that does not produce functional protein with IPTG induction used as a negative control? While the non-induced samples demonstrates whether IPTG induction is working properly or not, a template vector control would account for any residual effects from the expression system itself, which could impact following functional analyses.*

[Reply]: Firstly, I agree with the reviewer that the suggested grouping is undoubtedly safer. However, I believe our protein expression maps already provide sufficient evidence. The protein expression maps in many published articles are presented like ours and even worse than ours (see Lu YM, et al. Mol Biol Rep. doi:10.1007/s11033-009-9568-5.; Tararam CA, et al. Exp Parasitol. doi:10.1016/j.exppara.2010.04.008.; Song X, et al. Trop Med Hyg. doi:10.4269/ajtmh.15-0452.; Guo C, et al. Genes (Basel). doi:10.3390/genes9110559.; Song H, et al. Parasit Vectors. doi:10.1186/s13071-021-04596-7.). Moreover, the pET-28a vector is widely used globally, and it is generally accepted that induction of empty vectors (without the target gene insert) does not need to be proven repeatedly (see He L, et al. Parasitol Res. doi:10.1007/s00436-014-3935-4). In fact, an increasing number of journals no longer include protein expression maps in the main content of articles.

[Suggestion 15]: *Line 197 (Polyclonal antibody production): For the polyclonal antibody production, the details are extremely lacking. How much total volume was injected? Where specifically is "more points under the back"? How much blood was collected and where was it collected from? Additional details should be provided about the ELISA.*

[Reply]: We are sorry for this. We have supplemented the details of where the blood is collected from. As for the total injection amount, I think we have explained it very clearly. What we describe is 'The recombinant proteins B1 and B2 were used as immune antigens. 500 μg recombinant protein was mixed with Freund's complete adjuvant at 1:1 for emulsification. 250 μg protein was mixed with Freund's incomplete adjuvant at 1:1 in the second, third, and fourth times.'. If you are interested in the volume, the concentration of Annexin B1 is 0.45 mg/mL, and the concentration of Annexin B2 is 0.48 mg/mL. Regarding the specific location of 'more points under the back', we realized our previous expression might have been unclear, so we have revised it to 'Multiple subcutaneous injections at the dorsal site'. Nowadays, many articles provide very limited details on the preparation of polyclonal antibodies, or even none at all (see Guo C, et al. Genes (Basel). doi:10.3390/genes9110559.; Tararam CA, et al. Exp Parasitol. doi:10.1016/j.exppara.2010.04.008.).

As for the details of ELISA, we have added them to the article. Specific content can be found in lines 226-248.

[Suggestion 16]: *Line 206 (Immunohistochemistry): While reference is cited for the IHC, at least brief overview details should be provided since this is an important part of the manuscript.*

[Reply]: We revised this part according to the reviewers' comments. Specific content can be found in lines 251-260.

[Suggestion 17]: *Line 209 (Prothrombin time (PT) and activated partial thromboplastin time (APTT) of Annexin B1 and B2): How much venous blood was collected per volunteer? Does "10 healthy volunteers" refer to human subject? If so, ethical approvals for human sample collection and use should be explicitly stated. How was the blood handled between collection and testing? What volume of blood was used per test? What temperature was the experiment performed at?*

[Reply]: We have added all the missing details. More specific operation details can be found in our labeled kit instructions. Specific content can be found in lines 263-278.

We have included the ethical approval for the collection and use of human samples at the end of the manuscript, in section ‘Ethics approval and consent to participate’.

[Suggestion 18]: *Line 211: Was the serum pooled from all 10 volunteers? Were the three repeats of the assay technical replicates?*

[Reply]: We have added the missing details. Each sample was tested individually, rather than pooling 10 samples for detection. Each sample assay was technically replicated three times.

[Suggestion 19]: *Line 253 (Data analysis): The manuscript mentions the use of SPSS 22 statistical software, but does not specify statistical methods (e.g., t-test, ANOVA, etc.) used in each analysis.*

[Reply]: Since the statistical methods used for analyzing coagulation test data and plasma membrane repair test data were different, we did not describe our statistical methods in the ‘Data Analysis’ section. Instead, we included the statistical methods used in the respective method descriptions for each test.

[Suggestion 20]: *The figure legends throughout the results section lack essential details for clear and intuitive interpretation. The authors should provide more precise descriptions of experimental conditions, controls, and potential inconsistencies in the data.*

[Reply]: We have made some revisions to the figures, figure captions, and methods in accordance with the reviewers' comments. Should there still be any issues, please inform me in a timely manner. I would be extremely grateful.

[Suggestion 21]: *Line 267: Figure 1C and 1D are said to have “high purity”, but many background bands are clearly visible throughout the gels. While these are relatively highly purified, any amount of contaminant E. coli proteins in the assays may cause false positives in the assays. This is where having the empty vector control sample carried through the experiments would have been helpful, to show that after the same purification processes, the background proteins present are not enough to induce a signal.*

[Reply]: Thank you very much for your valuable comments and suggestions. We appreciate your attention to the potential issue of contamination in our protein purification process.We understand the importance of ensuring that any residual *E. coli* proteins do not contribute to false-positive results in our analysis. While we agree that

including a purified empty vector control could be informative, we have taken several alternative measures to address this concern:

Stringent Purification Protocol: We employed a highly specific and stringent purification protocol that includes multiple steps (e.g., affinity chromatography) to minimize the presence of contaminating proteins. The purity of the target protein was further confirmed by SDS-PAGE, which showed no detectable contaminating bands.

Negative Control in Downstream Analysis: In addition to the purification steps, we included negative controls in our downstream functional assays. These controls were performed without the addition of any purified protein, and the results demonstrated that the observed signals were specific to the target protein and not due to background contamination.

We believe that these measures collectively ensure the reliability and specificity of our experimental results. Including an empty vector control in the purification process would not add substantial value, given the high purity of our target protein and the stringent controls already in place. We hope this explanation addresses your concerns. If you have any further suggestions or require additional information, please let us know.

[Suggestion 22]: *Lines 259 and 269: In Figure 1E, why are the two proteins now different masses and higher masses than in the previous panels? They go from 35kDa (line 259) to 40 and 43 kDa (line 270). Previous studies estimated molecular weight of annexin B1 (PMID: 14766301 and 16857011) at ~38 kDa and B2 (PMID: 19455404) at 38.5 kDa. The authors should explain this discrepancy. Is it due to post-translational modifications, alternative splicing, or protein tagging? If so, this should be explicitly stated.*

[Reply]: This is an excellent question. The primary reason is that different expression vectors may carry fusion tags or other modifying elements. These elements are co-expressed with the target protein, thereby affecting the size of the final protein.

[Suggestion 23]: *Line 271: Is this supposed to be serum from infected pigs? Where did the pigs come from? Did the authors infect the pigs? If so, then very detailed methods need to be provided.*

[Reply]: Yes, this is indeed pig serum. More details can be found in section ‘Study animals’. Specific content can be found in lines 174-176.

[Suggestion 24]: *The Western blot (Figure 1F) shows two bands per protein (upper and lower bands for annexin B1 and B2). Is it a sign up protein degradation or presence of isoforms? The authors should investigate and clarify the reason for this band pattern.*

[Reply]: Firstly, we can rule out the possibility that the impurities are due to poor purification, as their locations are not the same. We speculate that the protein has undergone slight degradation, which is hard to avoid during the experiment. Moreover, what we used is not a monoclonal antibody but positive serum from porcine cysticercosis. We hope you can understand this situation. If you think this figure is not scientific and rigorous enough, please let us know in time. We would be very grateful.

[Suggestion 25]: *Line 294: In Figure 2, what is the negative control? A negative control using a purified sample from the empty vector control throughout the purification process would be more convincing.*

[Reply]: As described in the article, the negative control used rabbit negative serum. The negative serum exhibited an acceptable background level in the experiments, effectively ruling out the possibility of non-specific binding, thereby demonstrating the efficacy of the polyclonal antibody. Regarding the negative control mentioned by the reviewer, we have not been able to find any literature that employs a purified empty vector as a control group. We believe that rabbit negative serum is a more universal negative control. If you have any further suggestions or require additional information, please let us know.

[Suggestion 26]: *Annexin B1 has already been demonstrated as immunogen (PMID: 17349964). However, the authors does not reference this. The omission could mislead readers into thinking that this is novel finding. The authors should cite previous work and explain how their findings build upon or differ from existing studies.*

[Reply]: The reviewer's considerations are indeed comprehensive. However, it should be noted that our manuscript does not intend to convey such an implication, as we have not elaborated extensively on this topic. Moreover, the notion that a certain protein can serve as an immunogen is hardly a novel finding, given that the vast majority of proteins are capable of being used to generate polyclonal antibodies. We have already cited a sufficient number of references regarding these two proteins. Should the journal editor consider it necessary to make a statement, we will continue to provide additional information.

[Suggestion 27]: *Line 301: In Figs 2A and 2B, the "full uncropped" gel images file has these gels cropped to exactly the same degree as the figure. Figure 2B also appears to have very high contrast, and both have dark grey smears of binding. The full gels would need to be provided to evaluate any potential background binding / protein degradation.*

[Reply]: Thank you for raising this suggestion. To be honest, what we submitted were the complete gel images. During the transfer process, we only needed to ensure that the marker and the target proteins were successfully transferred from the gel to the nitrocellulose (NC) membrane. Therefore, we did not waste any additional materials. I don't understand what you are concerned about regarding the contrast. The bands are already so dark that I don't think any non-specific bands would be deliberately concealed by us.

[Suggestion 28]: *Line 310: There is a lack of proper citation, and this result appears redundant. Annexin B1 was previously detected in the tegument of Cysticercus cellulosae (PMID: 17936648).*

[Reply]: Thank you for raising this question. However, it should be noted that the referenced study was published a considerable time ago, and more critically, it only provided images taken at a magnification of 100×. This may lead readers to struggle in comprehending the results and even question their validity. In contrast, our images are of significantly higher quality and have successfully validated their findings. Therefore, I do not believe that our results are redundant. I will appropriately cite their results in the discussion section.

[Suggestion 29]: *Lines 315-318: Methods from Figures 3 and 4 need to be expanded (may be mentioned already).*

[Reply]: Yes, you have already mentioned it. We made the modification according to your suggestion.

[Suggestion 30]: *Line 315: In Fig 3, the settings of the microscope including brightness and contrast are visibly different between positive and negative controls. For a fair comparison, these should be kept as consistent as possible to evaluate background signal fairly. In addition, seeing some distinct tissues in the positive controls that are not stained, from different sections, would help to demonstrate the tissue-specific claims made more clearly. Scale bars should also be provided, and a positive control*

of a different marker with known tissue specificity would help to make the results more convincing. Scale bars should be shown in addition to the magnification being specified.

[Reply]: I can assure you that these images have not been altered in any way, including adjustments to contrast or brightness—they are the original images. Reducing the brightness or contrast of the negative control images would be of no benefit to me. In these results, it is quite evident that the negative controls were performed very well, with no non-specific staining observed. We speculate that the color discrepancy may be attributed to improper handling of the gum sealing sheets, which led to inconsistent white balance during photography. Additionally, the scale bars were not conspicuous due to the insufficient clarity of the images, but we have now resolved this issue.

[Suggestion 31]: *Line 322: Instead of comparing annexin B1 to annexin B2, a more relevant comparison would be annexin B1/B2 against appropriate negative controls.*

[Reply]: Indeed, comparing the experimental group with the control group is essential. We have continued to refine this aspect. However, comparing B1 and B2 with each other is also highly informative, and we intend to retain this part of the analysis. If you have any further suggestions or require additional information, please let us know.

[Suggestion 32]: *Line 325: Again, this general statement shouldn't be supported by a P value since it is describing all of the groups together. If a test was ran to support the overall trend, then it should be mentioned.*

[Reply]: Thank you for your suggestion. We have now removed the P-values.

[Suggestion 33]: *Line 326: This observation is not novel and was previously reported (PMID: 19455404).*

[Reply]: We are not the first to investigate the anticoagulant activity of these two proteins. However, I suspect that the reviewer might not have seen the discussion section when they raised this comment. We have actually discovered some results that were not previously reported by others. Specific content can be found in lines 533-540.

[Suggestion 34]: *Line 329: Testing this coagulation assay with up to 200μg/mL of a negative control protein like BSA would help to demonstrate the activity in Annexin (as performed in PMID:19455404). At those concentrations, coagulation may be disrupted just do to oversaturation of the mixture and physical blocking of proteins. These concentrations are also not physiologically relevant.*

[Reply]: We concur with your concern. Many studies have also included irrelevant proteins as controls (see Lu YM, et al. Mol Biol Rep. doi:10.1007/s11033-009-9568-5; de la Torre-Escudero E, et al. Vet Parasitol. doi:10.1016/j.vetpar.2011.08.013). Accordingly, we have added BSA (200 μg/mL) as a control group. Similar to the findings in other studies, BSA exhibited no anticoagulant activity. This further strengthens the reliability of our results.

[Suggestion 35]: *Line 360: The negative controls show significant activity, which raises concerns about their validity. Are they proper negative controls? Could the buffer composition, residual contaminants, or protein aggregation contribute to unexpected activity?*

[Reply]: This is a well-documented phenomenon. We came across a Chinese publication that mentioned 'Coagulation function is related to the degree of blood dilution. When the proportion of normal saline reaches 50%, the blood's coagulation ability begins to weaken. When the proportion of normal saline is 60%, the coagulation ability is severely affected.' in its conclusions, which is consistent with our results (see Zhuoqiang Wang, et al. Effects of dilution of blood with different amount of physiological saline in vitro on blood coagulability. Medical Journal of Chinese People's Liberation Army. 2008, (10): 1268-1271). Moreover, the differences between the normal saline group and the annexin group (200 μg/mL) in our study were extremely significant ($P < 0.0001$). Therefore, we can confidently rule out the possibility of 'unexpected activity'. When studying the coagulation function of annexin from *Schistosoma bovis*, the results of coagulation function in plasma diluted with buffer components were similar to ours (see de la Torre-Escudero E, et al. Vet Parasitol. doi:10.1016/j.vetpar.2011.08.013).

[Suggestion 36]: *Line 367: For Figure 5, the specific statistical test should be specified (T-tests?). The number of replicates and the error bars indicating standard error or deviation should be provided in the caption. Multiple test correction should be performed at least for the comparisons within each graph. Specify what "NS" on the X axis is in the caption.*

[Reply]: This was an oversight on our part. We have now supplemented the statistical tests used in the 'Prothrombin time (PT) and activated partial thromboplastin time (APTT) of Annexin B1 and B2'. Additionally, we mentioned that our data are presented as mean ± SD in the 'Data analysis'. We also noticed that the use of 'ns' and 'NS' might

have caused confusion. We have added the meaning of ‘NS’ as well as the number of replicates, as you suggested, to the caption of Figure 5.

[Suggestion 37]: *Line 368: For Figure 5, describe the positive and negative controls in the caption so that readers don’t have to find those details again in the methods. The concentration of those controls is critically important for evaluating the relative strength of Annexin anticoagulation, but they are not provided.*

[Reply]: We agree with your perspective. This approach indeed facilitates a clearer understanding of our figures for the readers. Therefore, we have transferred some of the explanations from the main text to the captions below the figures.

[Suggestion 38]: *Line 371: For the lipid binding assay, some positive and negative controls with known reactivity would be helpful to evaluate the system, in terms of background staining. Again here, negative controls (a purified sample from the empty vector control) would help.*

[Reply]: The reviewer's concerns are not without merit. However, we are not the first to employ this experimental method. Phospholipid-binding assays have been widely used in numerous studies without the inclusion of negative or positive controls (see Kim SC, et al. Plant Cell. doi:10.1105/tpc.18.00675; Nakamura Y, et al. Nat Commun. doi:10.1038/ncomms4553; Susila H, et al. Science. doi:10.1126/science.abh4054). Additionally, Annexin B1 has been previously demonstrated to possess phospholipid-binding capabilities (see Zhang Y, et al. Protein Expr Purif. doi:10.1016/j.pep.2003.11.025). Nevertheless, given that this aspect is closely related to our subsequent investigation on plasma membrane repair, we deemed it necessary to personally verify the authenticity of these results. If you have any further suggestions or require additional information, please let us know.

[Suggestion 39]: *The amount of annexin B1/B2 added to each assay is missing in the method section. The direct intensity comparison between annexin B1 and B2 may not be appropriate unless equal amounts of protein were used and quantified.*

[Reply]: We apologize for the omission. The details of these methods are available in the references we cited. We have chosen to omit them here to avoid redundancy and to respect originality. The literature mentions ‘Discard the blocking solution and incubate the membrane with 60 µg of recombinant His-FT or FT-StII protein in 5 mL of blocking solution at 4°C for 16 h with gentle agitation (10 rpm)’. So indeed, we did quantify the protein.

[Suggestion 40]: *Line 380: This may be better suited as supplemental results rather than a main finding, depending on its significance to the overall study conclusions. The authors should justify why this particular result is central to the study, or move it to the supplementary section.*

[Reply]: Firstly, the binding capacity of Annexin B2 to phosphatidylserine (PS) appears to be a novel finding, as no previous studies have validated this interaction to our knowledge. The potential roles of PS in coagulation and plasma membrane repair have been discussed in our manuscript. This result not only helps to substantiate our experimental findings but also provides valuable insights for future research into the detailed mechanisms of function. Given the novelty of this finding, we believe that these results merit inclusion in the main text of the article. However, if the editor deems that these results are better suited as supplementary data, we will certainly follow your recommendations.

[Suggestion 41]: *Line 385: If Figure 6C should be included as a main result, it may be more appropriate to combine it with Figure 7, which presents the same experimental context. The authors should ensure that figure panels are organized in a way that enhances logical flow and data interpretation.*

[Reply]: Thank you for your suggestions. We have revised the content accordingly.

[Suggestion 42]: *Line 394: As mentioned above, the authors should justify why plasma membrane of mammalian cells were used instead of the parasite's plasma membrane (due to technical difficulties to obtain the parasite's ones?). How doe findings in the mammalian plasma membrane relate to membrane repair mechanisms in Cysticercus cellulosae?*

[Reply]: Firstly, for tapeworms (such as *Taenia solium* and *Taenia saginata*), we have not yet succeeded in establishing stable cell lines from individual cells, which remains a significant challenge. We have not seen any published reports of successful attempts in this regard also. The structure of the plasma membrane is largely conserved across species, with glycolipids accounting for approximately 70% of the total lipid content in mammalian cells; the remaining 30% consists of cholesterol, sphingomyelin, and glycosphingolipids (see Leventis PA, Grinstein S. Annu Rev Biophys. doi:10.1146/annurev.biophys.093008.131234). The proportion of lipid types may vary depending on the tissue. Therefore, we have chosen the C2C12 cell line as a model to investigate the plasma membrane repair mechanisms of Taenia solium cysticerci. As is

well known, models are crucial in biomedical research, whether for elucidating disease mechanisms, drug development, or vaccine research, serving as an irreplaceable tool. In the absence of a mature cell line for *Cysticercus cellulosae,* it is essential to continue utilizing various models to lay the groundwork for more detailed validation in the future. If you have any further suggestions or require additional information, please let us know.

[Suggestion 43]: *Lines 423-429: Figures 7 and 8 could also be combined as four panels instead of separate figures. Captions for Fig 7 and 8 should be more descriptive, about what signal is being detected as green, what type of microscopy it is, what the cells are, etc. Were the microscope settings ensured to be identical for each run of the wounding assay?*

[Reply]: Following your suggestion, we have merged them into a single image. We have also added the "cells type" and "what signal is being detected as green" to the figure caption. Additionally, we have specified the microscope model in the Methods section and included a statement ensuring that the instrument settings are consistent for each run.

[Suggestion 44]: *In Figure 8, why wasn't a -Ca^{2+} group shown for the annexins? This would help show the difference in the red and blue in Fig 9A.*

[Reply]: As described in our manuscript, the plasma membrane repair process is highly dependent on Ca^{2+} signaling. In the absence of Ca^{2+}, the cell will not repair the plasma membrane. Therefore, their Ca^{2+}-free group is identical to Figure 7B. To avoid redundancy, we have omitted the set of images you mentioned. This is also the case in other studies (see Abreu-Blanco MT, et al. Bioarchitecture. doi:10.4161/bioa.1.3.17091; Boye TL, Maeda K, et al. Nat Commun. doi:10.1038/s41467-017-01743-6; Carmeille R, et al. Biochim Biophys Acta. doi:10.1016/j.bbamcr.2014.12.038; Sønder SL, et al. Sci Adv. doi:10.1126/sciadv.abg1969).

[Suggestion 45]: *Line 430: Figure 9, what is the statistical test performed here? What are the error bars? How many replicates were tested, and were they biological replicates or technical?*

[Reply]: The answers to these questions can be found in sections 'Plasma membrane repair kinetics upon laser injury in live cells' and 'Data analysis' of the manuscript. The statistical analysis used in this experiment involved first calculating the area under the curve (AUC) for each curve, followed by an unpaired Welch's t-test, which is consistent with the description in the manuscript (see Sønder SL, et al. Bio Protoc.

doi:10.21769/BioProtoc.4437). Each group consisted of 15 samples, with biological replicates.

[Suggestion 46]: *In Figure 8, the fluorescence intensity for the annexins with Ca2+ appears extremely low and barely detectable, so why is the value so high In Figure 9A and 9B compared to the degree observed in control (Figure 7 and Figure 9C)?*

[Reply]: Firstly, I believe it is normal for the fluorescence intensity in the $+Ca^{2+}$ of Annexin group to be lower than that in the control group, but it is not nearly undetectable. The discrepancy might be due to the small size of the images. By zooming in on the images, the green fluorescence can be more clearly observed.

Regarding the values in Figure 9, there are inherent differences between visual inspection by the human eye and quantitative measurements made using software. Additionally, these values represent the mean ± SD obtained from measurements of 15 cells. If you have any further suggestions or require additional information, please let us know.

[Suggestion 47]: *Line 546: are the panels in Figs 7 and 8 actually electron microscopy or is this just a typo? If electron microscopy was used, then substantial details should be provided about how that was performed.*

[Reply]: I'm terribly sorry. We don't understand the meaning of your question. We didn't find anything amiss with the figures. If you could provide a detailed explanation of your concerns, we would be happy to respond at any time.
