# Supplementary material for: Functional identification of Annexin B1 and Annexin B2 from Cysticercus cellulosae and their mechanism in plasma membrane repair
Source: PLoS Negl Trop Dis. 2025 Apr 17;19(4):e0013015. doi: 10.1371/journal.pntd.0013015 (PMC12005505; doi:10.1371/journal.pntd.0013015)
Supplement: Attachment — Submitted filename: Review_Final.docx [file pntd.0013015.s010.docx]

**Overview statement**

This study aimed to investigate the molecular functions of recombinant annexin B1 and B2 proteins from *Cysticercus cellulosae* with various assay approaches. The authors expressed and purified annexin B1 and B2 proteins and examined their immunogenicity, histological distribution, anticoagulant activity, phospholipid binding activity, and role in plasma membrane repairs.

While the study presented some relevant findings, the manuscript lacks sufficient methodological details, includes redundant results that have already been reported in previous studies, and contains flaws in data interpretation. In addition, the writing requires substantial revision to improve clarity and coherence in English.

**Specific comments**

- Line 32: The immunohistochemistry results indicate the localization of annexin B1 and B2 but do not provide direct evidence supporting their proposed functions, such as maintaining the worm's integrity, assisting movement, and nutrient absorption and digestion. Additional functional assays will be required to substantiate these claims.

- Line 40: Similar concerns apply to the interpretation of the plasma membrane repair test. The study should clarify the structural and compositional similarities and differences between the parasite’s plasma membrane and mammalian ones. How do these factors influence the proposed membrane repair function of annexin B1 and B2 in *Cysticercus cellulosae*?

- Line 44: The claim that annexin B1 and B2 contributes to immune evasion or inhibition of host immunity lacks direct supporting evidence. The authors should specify whether these proteins have multiple functions, including structural maintenance, facilitation of parasite migration, nutrient digestion and absorption, and immune evasion and provide experimental validation or references to support each role.

The introduction should provide a clear rationale for why the authors specifically pursued the anticoagulant and plasma membrane repair functions of annexins based on prior studies and literature. If certain findings are not novel, the authors should cite papers and acknowledge prior work accordingly.

- Line 51: This statement is logically flawed. The fact that a disease is classified as “neglected” does not imply that the pathogen has exceptional immune evasion abilities. In other words, a pathogen’s ability to evade the host immune system is not a direct consequence of the disease itself. The authors should rephrase this statement with supporting evidence and references.

- Lines 56-57: These are example sentences which need revisions. Sentences should not begin with “And”.

- Line 79: The authors state that the mechanisms of invasion and immune escape are not well understood, yet the rest of paragraph describes how the parasites modulate the host immune response for survival in the hosts. The contradiction needs to be addressed, either by clarifying the unknown aspects of immune evasion or refining the statement to acknowledge existing knowledge.

- Line 107: The degree of conservation of the proteins should be clarified. Are the authors referring to functional conservation, sequence conservations, or both? What is the level of conservation, across eukaryotes, or more broadly prokaryotes and eukaryotes?

- Line 110: The phrase “the unique and highly conserved C-terminal” is contradictory. If a sequence is highly conserved, it implies similarity across species or homologs, whereas “unique” suggests distinctiveness. The authors should clarify whether they mean that the C-terminal is conserved within the annexin family while having functional specificity.

- Line 126: The authors should specify how the expression of ANX A1 protein in parasitic vacuoles supports its potential as a therapeutic target. Additional supporting evidence or mechanistic details are needed to justify this claim.

- Lines 146-154: The overall conclusion is not well-supported by observations and/or data presented in the study. The authors make broad claims about annexin B1 and B2’s roles in parasite invasions and immune evasions without providing sufficient experimental validation. In addition, the section lacks coherence, as it presents multiple functional roles without a clear logical connection between them. The authors should refine their conclusions to align more closely with the data presented and clarify the mechanistic links between annexin functions.

The methods sections requires substantial revisions and additional detail to allow for proper evaluation and reproducibility. Key experimental parameters, such as specific protocols, reagent/sample concentrations, sample sizes, and statistical analyses, are either missing or insufficiently described. The authors should provide greater clarity on experimental controls, data normalization methods, and any modifications to standard protocols. Without these details, it is difficult to assess the rigor and reliability of the study.

- Line 156: The “Study animals” subsection only says “2 ±0.2 kg adult female rabbits.” The authors should provide more details such as the rabbits’ age, housing conditions, feeding regimen, and ethical approvals for animal use. Furthermore, while the study includes pig samples, the manuscript does not describe their source, handling, or relevant experimental details. These omissions need to be addressed to ensure transparency and reproducibility.

- Line 158 (Expression of annexin B1 and annexin B2): Where were the target gene sequences retrieved from? Is there any transcriptomic evidence that there may be multiple isoforms of the genes? What primers were used? What was the template material (e.g., total RNA or cDNA) for amplification? Where were the worms sourced from? How many worms were used?

- Line 173 (Purification of annexin B1 and annexin B2): The group without IPTG was used as a negative control, but why wasn’t the template empty pET-28a(+) vector (without the insert) or a vector with a gene insert that does not produce functional protein with IPTG induction used as a negative control? While the non-induced samples demonstrates whether IPTG induction is working properly or not, a template vector control would account for any residual effects from the expression system itself, which could impact following functional analyses.

- Line 197 (Polyclonal antibody production): For the polyclonal antibody production, the details are extremely lacking. How much total volume was injected? Where specifically is “more points under the back”? How much blood was collected and where was it collected from? Additional details should be provided about the ELISA.

- Line 206 (Immunohistochemistry): While reference is cited for the IHC, at least brief overview details should be provided since this is an important part of the manuscript.

- Line 209 (Prothrombin time (PT) and activated partial thromboplastin time (APTT) of Annexin B1 and B2): How much venous blood was collected per volunteer? Does “10 healthy volunteers” refer to human subject? If so, ethical approvals for human sample collection and use should be explicitly stated. How was the blood handled between collection and testing? What volume of blood was used per test? What temperature was the experiment performed at?

- Line 211: Was the serum pooled from all 10 volunteers? Were the three repeats of the assay technical replicates?

- Line 253 (Data analysis): The manuscript mentions the use of SPSS 22 statistical software, but does not specify statistical methods (e.g., t-test, ANOVA, etc.) used in each analysis.

The figure legends throughout the results section lack essential details for clear and intuitive interpretation. The authors should provide more precise descriptions of experimental conditions, controls, and potential inconsistencies in the data.

- Line 267: Figure 1C and 1D are said to have “high purity”, but many background bands are clearly visible throughout the gels. While these are relatively highly purified, any amount of contaminant E. coli proteins in the assays may cause false positives in the assays. This is where having the empty vector control sample carried through the experiments would have been helpful, to show that after the same purification processes, the background proteins present are not enough to induce a signal.

- Lines 259 and 269: In Figure 1E, why are the two proteins now different masses and higher masses than in the previous panels? They go from 35kDa (line 259) to 40 and 43 kDa (line 270). Previous studies estimated molecular weight of annexin B1 (PMID: 14766301 and 16857011) at ~38 kDa and B2 (PMID: 19455404) at 38.5 kDa. The authors should explain this discrepancy. Is it due to post-translational modifications, alternative splicing, or protein tagging? If so, this should be explicitly stated.

- Line 271: Is this supposed to be serum from infected pigs? Where did the pigs come from? Did the authors infect the pigs? If so, then very detailed methods need to be provided.

The Western blot (Figure 1F) shows two bands per protein (upper and lower bands for annexin B1 and B2). Is it a sign up protein degradation or presence of isoforms? The authors should investigate and clarify the reason for this band pattern.

- Line 294: In Figure 2, what is the negative control? A negative control using a purified sample from the empty vector control throughout the purification process would be more convincing.

Annexin B1 has already been demonstrated as immunogen (PMID: 17349964). However, the authors does not reference this. The omission could mislead readers into thinking that this is novel finding. The authors should cite previous work and explain how their findings build upon or differ from existing studies.

- Line 301: In Figs 2A and 2B, the “full uncropped” gel images file has these gels cropped to exactly the same degree as the figure. Figure 2B also appears to have very high contrast, and both have dark grey smears of binding. The full gels would need to be provided to evaluate any potential background binding / protein degradation.

- Line 310: There is a lack of proper citation, and this result appears redundant. Annexin B1 was previously detected in the tegument of *Cysticercus cellulosae* (PMID: 17936648).

- Lines 315-318: Methods from Figures 3 and 4 need to be expanded (may be mentioned already).

- Line 315: In Fig 3, the settings of the microscope including brightness and contrast are visibly different between positive and negative controls. For a fair comparison, these should be kept as consistent as possible to evaluate background signal fairly. In addition, seeing some distinct tissues in the positive controls that are not stained, from different sections, would help to demonstrate the tissue-specific claims made more clearly. Scale bars should also be provided, and a positive control of a different marker with known tissue specificity would help to make the results more convincing. Scale bars should be shown in addition to the magnification being specified.

- Line 322: Instead of comparing annexin B1 to annexin B2, a more relevant comparison would be annexin B1/B2 against appropriate negative controls.

- Line 325: Again, this general statement shouldn’t be supported by a P value since it is describing all of the groups together. If a test was ran to support the overall trend, then it should be mentioned.

- Line 326: This observation is not novel and was previously reported (PMID: 19455404).

- Line 329: Testing this coagulation assay with up to 200µg/mL of a negative control protein like BSA would help to demonstrate the activity in Annexin (as performed in PMID:19455404). At those concentrations, coagulation may be disrupted just do to oversaturation of the mixture and physical blocking of proteins. These concentrations are also not physiologically relevant.

- Line 360: The negative controls show significant activity, which raises concerns about their validity. Are they proper negative controls? Could the buffer composition, residual contaminants, or protein aggregation contribute to unexpected activity?

- Line 367: For Figure 5, the specific statistical test should be specified (T-tests?). The number of replicates and the error bars indicating standard error or deviation should be provided in the caption. Multiple test correction should be performed at least for the comparisons within each graph. Specify what “NS” on the X axis is in the caption.

- Line 368: For Figure 5, describe the positive and negative controls in the caption so that readers don’t have to find those details again in the methods. The concentration of those controls is critically important for evaluating the relative strength of Annexin anticoagulation, but they are not provided.

- Line 371: For the lipid binding assay, some positive and negative controls with known reactivity would be helpful to evaluate the system, in terms of background staining. Again here, negative controls (a purified sample from the empty vector control) would help.

More biological replicates are necessary to ensure statistical robustness.

The amount of annexin B1/B2 added to each assay is missing in the method section. The direct intensity comparison between annexin B1 and B2 may not be appropriate unless equal amounts of protein were used and quantified.

- Line 380: This may be better suited as supplemental results rather than a main finding, depending on its significance to the overall study conclusions. The authors should justify why this particular result is central to the study, or move it to the supplementary section.

- Line 385: If Figure 6C should be included as a main result, it may be more appropriate to combine it with Figure 7, which presents the same experimental context. The authors should ensure that figure panels are organized in a way that enhances logical flow and data interpretation.

- Line 394: As mentioned above, the authors should justify why plasma membrane of mammalian cells were used instead of the parasite’s plasma membrane (due to technical difficulties to obtain the parasite’s ones?). How doe findings in the mammalian plasma membrane relate to membrane repair mechanisms in *Cysticercus cellulosae*?

- Lines 423-429: Figures 7 and 8 could also be combined as four panels instead of separate figures. Captions for Fig 7 and 8 should be more descriptive, about what signal is being detected as green, what type of microscopy it is, what the cells are, etc.

Were the microscope settings ensured to be identical for each run of the wounding assay?

In Figure 8, why wasn’t a -Ca2+ group shown for the annexins? This would help show the difference in the red and blue in Fig 9A.

- Line 430: Figure 9, what is the statistical test performed here? What are the error bars? How many replicates were tested, and were they biological replicates or technical?

- In Figure 8, the fluorescence intensity for the annexins with Ca2+ appears extremely low and barely detectable, so why is the value so high In Figure 9A and 9B compared to the degree observed in control (Figure 7 and Figure 9C)?

Overall, the discussion section requires substantial revision due to lack of proper citations, inappropriate interpretation of results, lack of coherence and unnecessary information.

- Line 546: are the panels in Figs 7 and 8 actually electron microscopy or is this just a typo? If electron microscopy was used, then substantial details should be provided about how that was performed.
